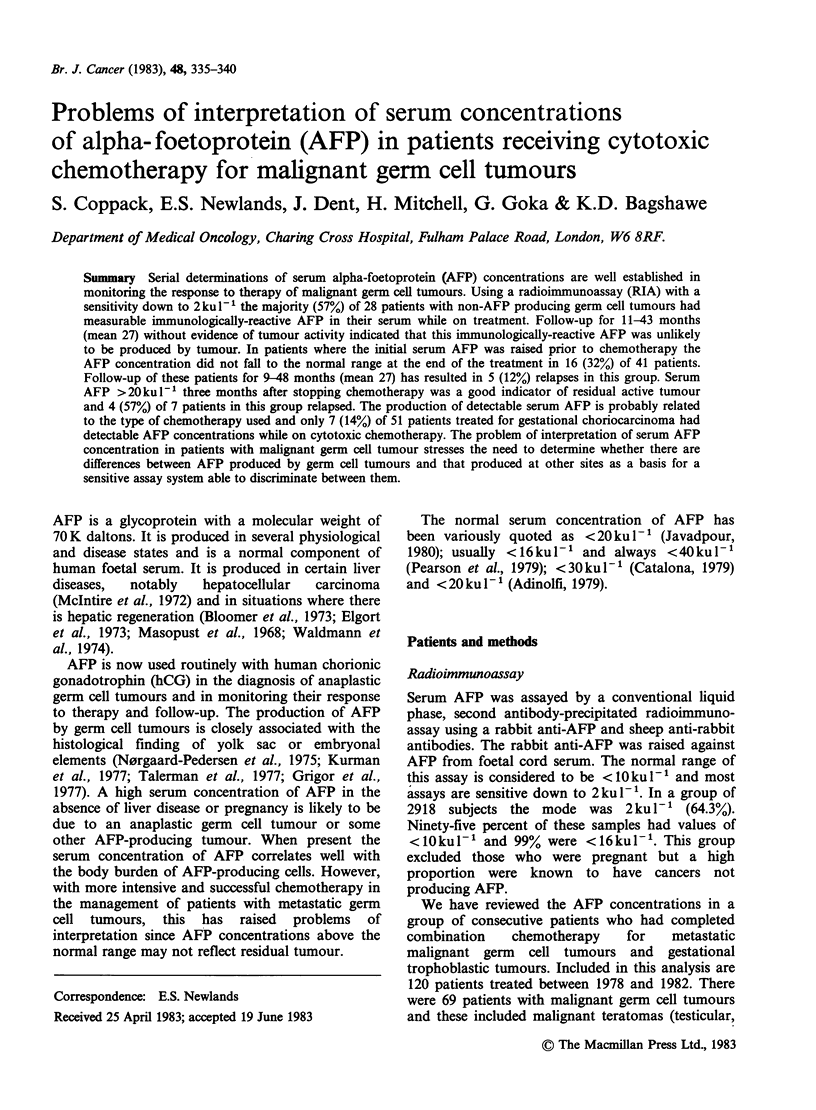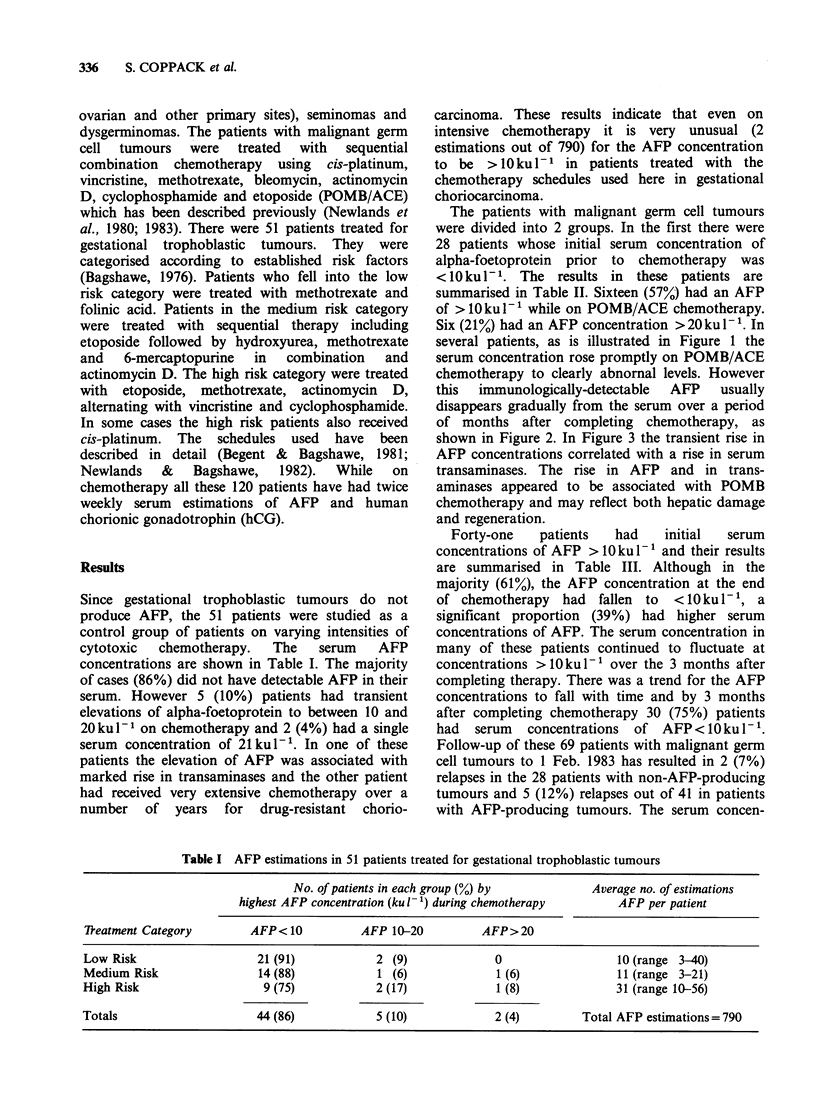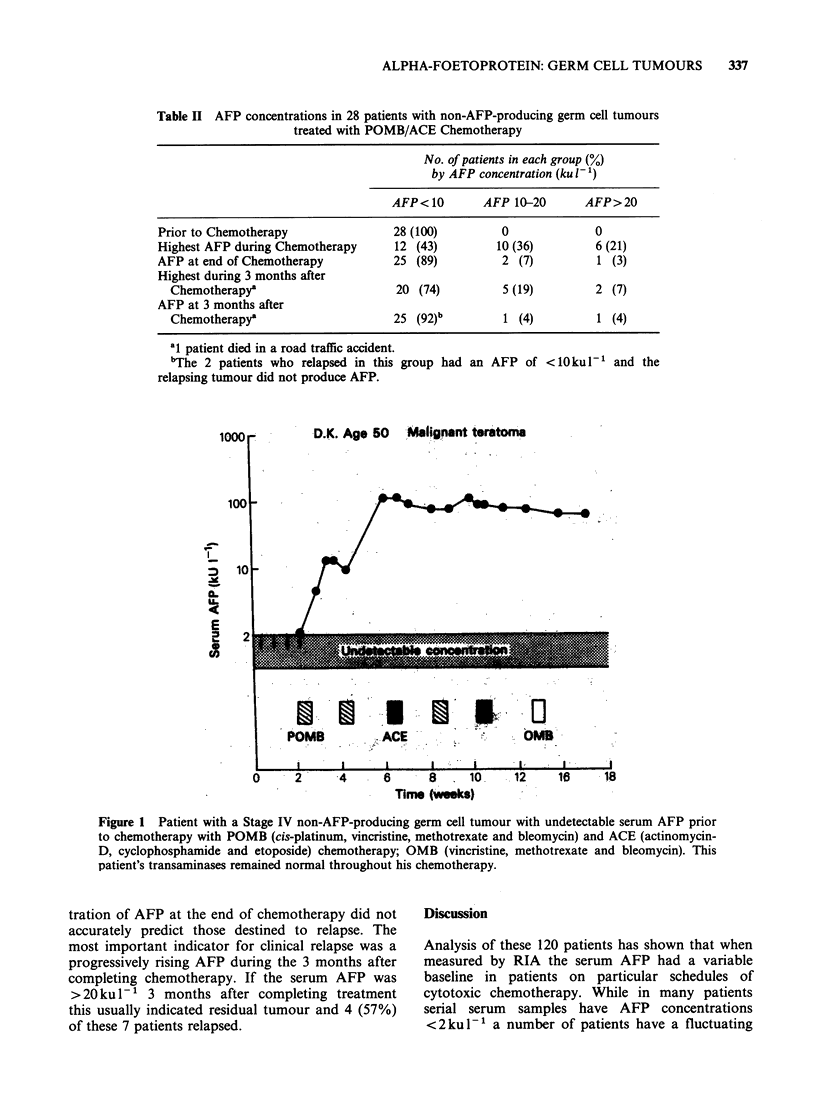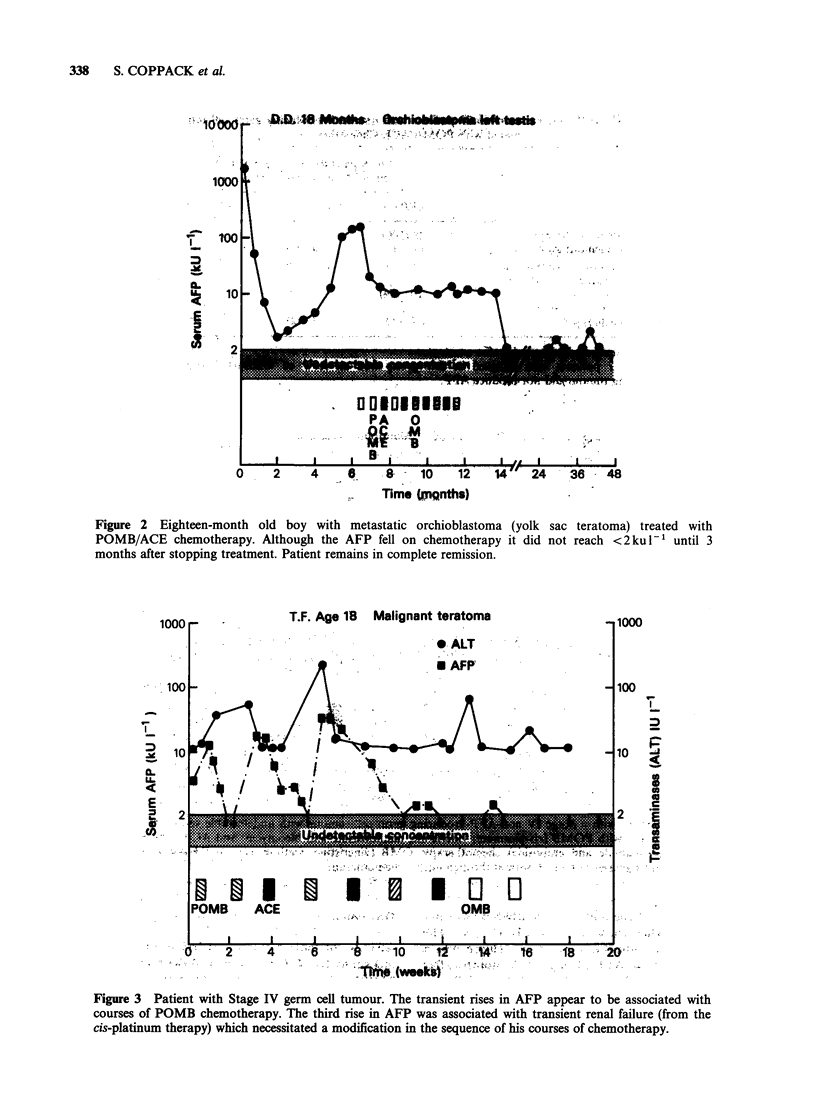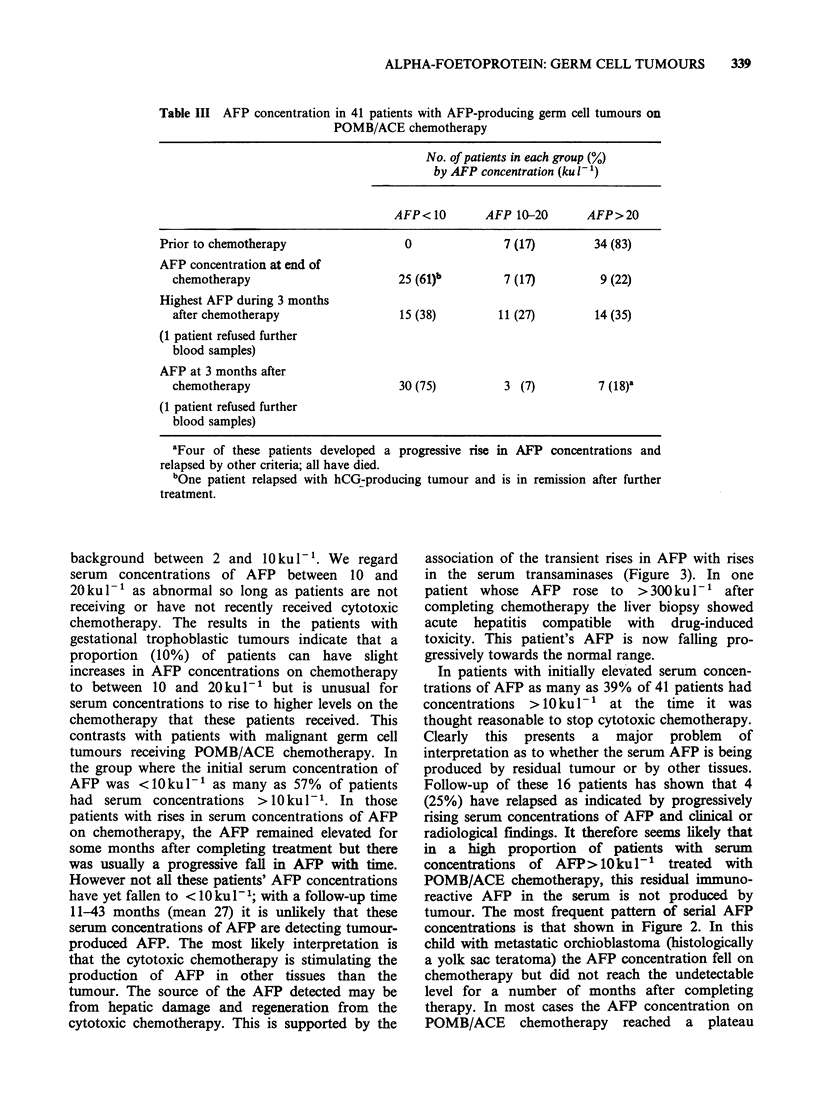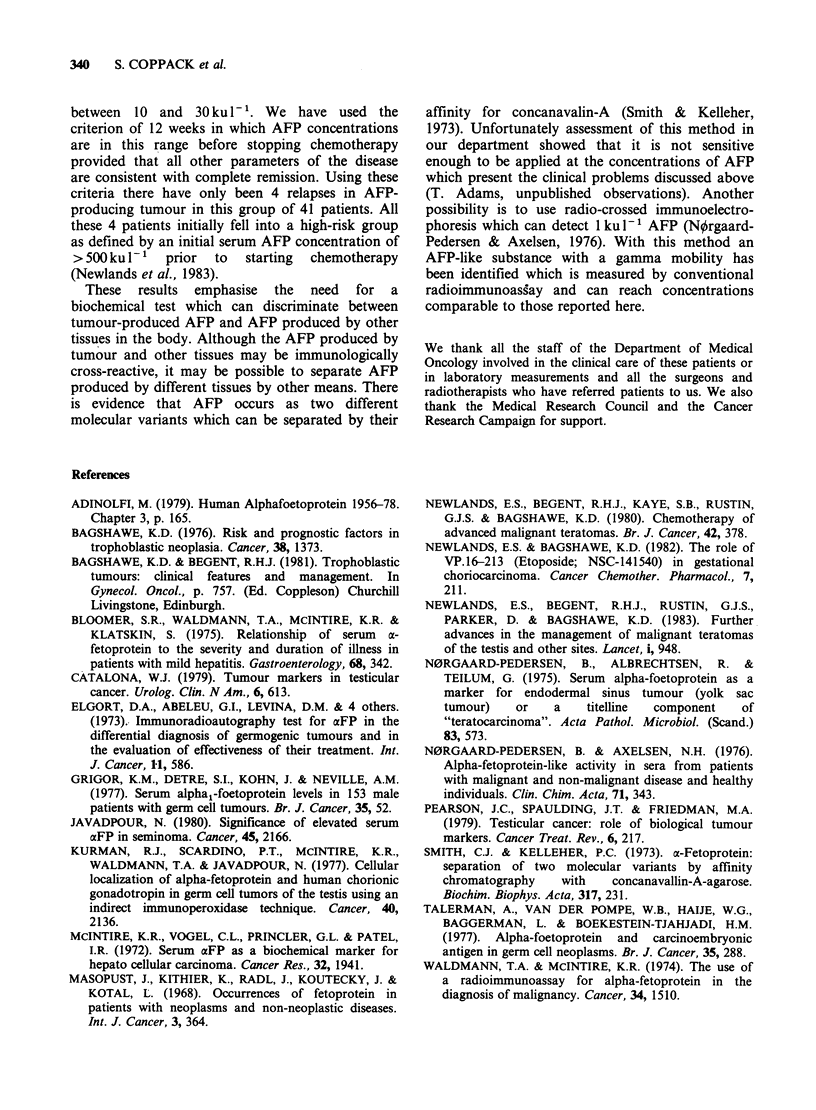# Problems of interpretation of serum concentrations of alpha-foetoprotein (AFP) in patients receiving cytotoxic chemotherapy for malignant germ cell tumours.

**DOI:** 10.1038/bjc.1983.197

**Published:** 1983-09

**Authors:** S. Coppack, E. S. Newlands, J. Dent, H. Mitchell, G. Goka, K. D. Bagshawe

## Abstract

Serial determinations of serum alpha-foetoprotein (AFP) concentrations are well established in monitoring the response to therapy of malignant germ cell tumours. Using a radioimmunoassay (RIA) with a sensitivity down to 2kul-1 the majority (57%) of 28 patients with non-AFP producing germ cell tumours had measurable immunologically-reactive AFP in their serum while on treatment. Follow-up for 11-43 months (mean 27) without evidence of tumour activity indicated that this immunologically-reactive AFP was unlikely to be produced by tumour. In patients where the initial serum AFP was raised prior to chemotherapy the AFP concentration did not fall to the normal range at the end of the treatment in 16 (32%) of 41 patients. Follow-up of these patients for 9-48 months (mean 27) has resulted in 5 (12%) relapses in this group. Serum AFP greater than 20kul-1 three months after stopping chemotherapy was a good indicator of residual active tumour and 4 (57%) of 7 patients in this group relapsed. The production of detectable serum AFP is probably related to the type of chemotherapy used and only 7 (14%) of 51 patients treated for gestational choriocarcinoma had detectable AFP concentrations while on cytotoxic chemotherapy. The problem of interpretation of serum AFP concentration in patients with malignant germ cell tumour stresses the need to determine whether there are differences between AFP produced by germ cell tumours and that produced at other sites as a basis for a sensitive assay system able to discriminate between them.


*Br. J. Cancer* (1983), **48**, 335–340

### Problems of interpretation of serum concentrations of alpha-foetoprotein (AFP) in patients receiving cytotoxic chemotherapy for malignant germ cell tumours

S. Coppack, E.S. Newlands, J. Dent, H. Mitchell, G. Goka & K.D. Bagshawe

*Department of Medical Oncology, Charing Cross Hospital, Fulham Palace Road, London, W6 8RF.*

**Summary** Serial determinations of serum alpha-foetoprotein (AFP) concentrations are well established in monitoring the response to therapy of malignant germ cell tumours. Using a radioimmunoassay (RIA) with a sensitivity down to $2\,\mathrm{ku\,l^{-1}}$ the majority (57%) of 28 patients with non-AFP producing germ cell tumours had measurable immunologically-reactive AFP in their serum while on treatment. Follow-up for 11–43 months (mean 27) without evidence of tumour activity indicated that this immunologically-reactive AFP was unlikely to be produced by tumour. In patients where the initial serum AFP was raised prior to chemotherapy the AFP concentration did not fall to the normal range at the end of the treatment in 16 (32%) of 41 patients. Follow-up of these patients for 9–48 months (mean 27) has resulted in 5 (12%) relapses in this group. Serum AFP $>20\,\mathrm{ku\,l^{-1}}$ three months after stopping chemotherapy was a good indicator of residual active tumour and 4 (57%) of 7 patients in this group relapsed. The production of detectable serum AFP is probably related to the type of chemotherapy used and only 7 (14%) of 51 patients treated for gestational choriocarcinoma had detectable AFP concentrations while on cytotoxic chemotherapy. The problem of interpretation of serum AFP concentration in patients with malignant germ cell tumour stresses the need to determine whether there are differences between AFP produced by germ cell tumours and that produced at other sites as a basis for a sensitive assay system able to discriminate between them.

AFP is a glycoprotein with a molecular weight of 70 K daltons. It is produced in several physiological and disease states and is a normal component of human foetal serum. It is produced in certain liver diseases, notably hepatocellular carcinoma (McIntire *et al.*, 1972) and in situations where there is hepatic regeneration (Bloomer *et al.*, 1973; Elgort *et al.*, 1973; Masopust *et al.*, 1968; Waldmann *et al.*, 1974).

AFP is now used routinely with human chorionic gonadotrophin (hCG) in the diagnosis of anaplastic germ cell tumours and in monitoring their response to therapy and follow-up. The production of AFP by germ cell tumours is closely associated with the histological finding of yolk sac or embryonal elements (Nørgaard-Pedersen *et al.*, 1975; Kurman *et al.*, 1977; Talerman *et al.*, 1977; Grigor *et al.*, 1977). A high serum concentration of AFP in the absence of liver disease or pregnancy is likely to be due to an anaplastic germ cell tumour or some other AFP-producing tumour. When present the serum concentration of AFP correlates well with the body burden of AFP-producing cells. However, with more intensive and successful chemotherapy in the management of patients with metastatic germ cell tumours, this has raised problems of interpretation since AFP concentrations above the normal range may not reflect residual tumour.

Correspondence: E.S. Newlands

Received 25 April 1983; accepted 19 June 1983

The normal serum concentration of AFP has been variously quoted as $<20\,\mathrm{ku\,l^{-1}}$ (Javadpour, 1980); usually $<16\,\mathrm{ku\,l^{-1}}$ and always $<40\,\mathrm{ku\,l^{-1}}$ (Pearson *et al.*, 1979); $<30\,\mathrm{ku\,l^{-1}}$ (Catalona, 1979) and $<20\,\mathrm{ku\,l^{-1}}$ (Adinolfi, 1979).

### Patients and methods

### *Radioimmunoassay*

Serum AFP was assayed by a conventional liquid phase, second antibody-precipitated radioimmunoassay using a rabbit anti-AFP and sheep anti-rabbit antibodies. The rabbit anti-AFP was raised against AFP from foetal cord serum. The normal range of this assay is considered to be $<10\,\mathrm{ku\,l^{-1}}$ and most assays are sensitive down to $2\,\mathrm{ku\,l^{-1}}$. In a group of 2918 subjects the mode was $2\,\mathrm{ku\,l^{-1}}$ (64.3%). Ninety-five percent of these samples had values of $<10\,\mathrm{ku\,l^{-1}}$ and 99% were $<16\,\mathrm{ku\,l^{-1}}$. This group excluded those who were pregnant but a high proportion were known to have cancers not producing AFP.

We have reviewed the AFP concentrations in a group of consecutive patients who had completed combination chemotherapy for metastatic malignant germ cell tumours and gestational trophoblastic tumours. Included in this analysis are 120 patients treated between 1978 and 1982. There were 69 patients with malignant germ cell tumours and these included malignant teratomas (testicular,

© The Macmillan Press Ltd., 1983

ovarian and other primary sites), seminomas and dysgerminomas. The patients with malignant germ cell tumours were treated with sequential combination chemotherapy using *cis*-platinum, vincristine, methotrexate, bleomycin, actinomycin D, cyclophosphamide and etoposide (POMB/ACE) which has been described previously (Newlands *et al.*, 1980; 1983). There were 51 patients treated for gestational trophoblastic tumours. They were categorised according to established risk factors (Bagshawe, 1976). Patients who fell into the low risk category were treated with methotrexate and folinic acid. Patients in the medium risk category were treated with sequential therapy including etoposide followed by hydroxyurea, methotrexate and 6-mercaptopurine in combination and actinomycin D. The high risk category were treated with etoposide, methotrexate, actinomycin D, alternating with vincristine and cyclophosphamide. In some cases the high risk patients also received *cis*-platinum. The schedules used have been described in detail (Begent & Bagshawe, 1981; Newlands & Bagshawe, 1982). While on chemotherapy all these 120 patients have had twice weekly serum estimations of AFP and human chorionic gonadotrophin (hCG).

### Results

Since gestational trophoblastic tumours do not produce AFP, the 51 patients were studied as a control group of patients on varying intensities of cytotoxic chemotherapy. The serum AFP concentrations are shown in Table I. The majority of cases (86%) did not have detectable AFP in their serum. However 5 (10%) patients had transient elevations of alpha-foetoprotein to between 10 and $20\,ku\,l^{-1}$ on chemotherapy and 2 (4%) had a single serum concentration of $21\,ku\,l^{-1}$. In one of these patients the elevation of AFP was associated with marked rise in transaminases and the other patient had received very extensive chemotherapy over a number of years for drug-resistant choriocarcinoma. These results indicate that even on intensive chemotherapy it is very unusual (2 estimations out of 790) for the AFP concentration to be $>10\,ku\,l^{-1}$ in patients treated with the chemotherapy schedules used here in gestational choriocarcinoma.

The patients with malignant germ cell tumours were divided into 2 groups. In the first there were 28 patients whose initial serum concentration of alpha-foetoprotein prior to chemotherapy was $<10\,ku\,l^{-1}$. The results in these patients are summarised in Table II. Sixteen (57%) had an AFP of $>10\,ku\,l^{-1}$ while on POMB/ACE chemotherapy. Six (21%) had an AFP concentration $>20\,ku\,l^{-1}$. In several patients, as is illustrated in Figure 1 the serum concentration rose promptly on POMB/ACE chemotherapy to clearly abnornal levels. However this immunologically-detectable AFP usually disappears gradually from the serum over a period of months after completing chemotherapy, as shown in Figure 2. In Figure 3 the transient rise in AFP concentrations correlated with a rise in serum transaminases. The rise in AFP and in transaminases appeared to be associated with POMB chemotherapy and may reflect both hepatic damage and regeneration.

Forty-one patients had initial serum concentrations of AFP $>10\,ku\,l^{-1}$ and their results are summarised in Table III. Although in the majority (61%), the AFP concentration at the end of chemotherapy had fallen to $<10\,ku\,l^{-1}$, a significant proportion (39%) had higher serum concentrations of AFP. The serum concentration in many of these patients continued to fluctuate at concentrations $>10\,ku\,l^{-1}$ over the 3 months after completing therapy. There was a trend for the AFP concentrations to fall with time and by 3 months after completing chemotherapy 30 (75%) patients had serum concentrations of AFP $<10\,ku\,l^{-1}$. Follow-up of these 69 patients with malignant germ cell tumours to 1 Feb. 1983 has resulted in 2 (7%) relapses in the 28 patients with non-AFP-producing tumours and 5 (12%) relapses out of 41 in patients with AFP-producing tumours. The serum concen-

**Table I** AFP estimations in 51 patients treated for gestational trophoblastic tumours

| | *No. of patients in each group (%) by highest AFP concentration ($ku\,l^{-1}$) during chemotherapy* | | | *Average no. of estimations AFP per patient* |
|---|---|---|---|---|
| *Treatment Category* | *AFP* < 10 | *AFP* 10–20 | *AFP* > 20 | |
| Low Risk | 21 (91) | 2 (9) | 0 | 10 (range 3–40) |
| Medium Risk | 14 (88) | 1 (6) | 1 (6) | 11 (range 3–21) |
| High Risk | 9 (75) | 2 (17) | 1 (8) | 31 (range 10–56) |
| Totals | 44 (86) | 5 (10) | 2 (4) | Total AFP estimations = 790 |

**Table II** AFP concentrations in 28 patients with non-AFP-producing germ cell tumours treated with POMB/ACE Chemotherapy

| | *No. of patients in each group (%) by AFP concentration* ($ku\,l^{-1}$) | | |
|---|---|---|---|
| | *AFP* < 10 | *AFP* 10–20 | *AFP* > 20 |
| Prior to Chemotherapy | 28 (100) | 0 | 0 |
| Highest AFP during Chemotherapy | 12 (43) | 10 (36) | 6 (21) |
| AFP at end of Chemotherapy | 25 (89) | 2 (7) | 1 (3) |
| Highest during 3 months after Chemotherapy[a] | 20 (74) | 5 (19) | 2 (7) |
| AFP at 3 months after Chemotherapy[a] | 25 (92)[b] | 1 (4) | 1 (4) |

[a]1 patient died in a road traffic accident.
[b]The 2 patients who relapsed in this group had an AFP of $<10\,ku\,l^{-1}$ and the relapsing tumour did not produce AFP.

**Figure 1** Patient with a Stage IV non-AFP-producing germ cell tumour with undetectable serum AFP prior to chemotherapy with POMB (*cis*-platinum, vincristine, methotrexate and bleomycin) and ACE (actinomycin-D, cyclophosphamide and etoposide) chemotherapy; OMB (vincristine, methotrexate and bleomycin). This patient's transaminases remained normal throughout his chemotherapy.

tration of AFP at the end of chemotherapy did not accurately predict those destined to relapse. The most important indicator for clinical relapse was a progressively rising AFP during the 3 months after completing chemotherapy. If the serum AFP was $>20\,ku\,l^{-1}$ 3 months after completing treatment this usually indicated residual tumour and 4 (57%) of these 7 patients relapsed.

### Discussion

Analysis of these 120 patients has shown that when measured by RIA the serum AFP had a variable baseline in patients on particular schedules of cytotoxic chemotherapy. While in many patients serial serum samples have AFP concentrations $<2\,ku\,l^{-1}$ a number of patients have a fluctuating

**Figure 2** Eighteen-month old boy with metastatic orchioblastoma (yolk sac teratoma) treated with POMB/ACE chemotherapy. Although the AFP fell on chemotherapy it did not reach $<2\,\mathrm{ku\,l^{-1}}$ until 3 months after stopping treatment. Patient remains in complete remission.

**Figure 3** Patient with Stage IV germ cell tumour. The transient rises in AFP appear to be associated with courses of POMB chemotherapy. The third rise in AFP was associated with transient renal failure (from the *cis*-platinum therapy) which necessitated a modification in the sequence of his courses of chemotherapy.

**Table III** AFP concentration in 41 patients with AFP-producing germ cell tumours on POMB/ACE chemotherapy

| | *No. of patients in each group (%) by AFP concentration* ($ku\,l^{-1}$) | | |
|---|---|---|---|
| | *AFP*<10 | *AFP* 10–20 | *AFP*>20 |
| Prior to chemotherapy | 0 | 7 (17) | 34 (83) |
| AFP concentration at end of chemotherapy | 25 (61)[b] | 7 (17) | 9 (22) |
| Highest AFP during 3 months after chemotherapy | 15 (38) | 11 (27) | 14 (35) |
| (1 patient refused further blood samples) | | | |
| AFP at 3 months after chemotherapy | 30 (75) | 3 (7) | 7 (18)[a] |
| (1 patient refused further blood samples) | | | |

[a]Four of these patients developed a progressive rise in AFP concentrations and relapsed by other criteria; all have died.

[b]One patient relapsed with hCG-producing tumour and is in remission after further treatment.

background between 2 and $10\,ku\,l^{-1}$. We regard serum concentrations of AFP between 10 and $20\,ku\,l^{-1}$ as abnormal so long as patients are not receiving or have not recently received cytotoxic chemotherapy. The results in the patients with gestational trophoblastic tumours indicate that a proportion (10%) of patients can have slight increases in AFP concentrations on chemotherapy to between 10 and $20\,ku\,l^{-1}$ but is unusual for serum concentrations to rise to higher levels on the chemotherapy that these patients received. This contrasts with patients with malignant germ cell tumours receiving POMB/ACE chemotherapy. In the group where the initial serum concentration of AFP was $<10\,ku\,l^{-1}$ as many as 57% of patients had serum concentrations $>10\,ku\,l^{-1}$. In those patients with rises in serum concentrations of AFP on chemotherapy, the AFP remained elevated for some months after completing treatment but there was usually a progressive fall in AFP with time. However not all these patients' AFP concentrations have yet fallen to $<10\,ku\,l^{-1}$; with a follow-up time 11–43 months (mean 27) it is unlikely that these serum concentrations of AFP are detecting tumour-produced AFP. The most likely interpretation is that the cytotoxic chemotherapy is stimulating the production of AFP in other tissues than the tumour. The source of the AFP detected may be from hepatic damage and regeneration from the cytotoxic chemotherapy. This is supported by the association of the transient rises in AFP with rises in the serum transaminases (Figure 3). In one patient whose AFP rose to $>300\,ku\,l^{-1}$ after completing chemotherapy the liver biopsy showed acute hepatitis compatible with drug-induced toxicity. This patient's AFP is now falling progressively towards the normal range.

In patients with initially elevated serum concentrations of AFP as many as 39% of 41 patients had concentrations $>10\,ku\,l^{-1}$ at the time it was thought reasonable to stop cytotoxic chemotherapy. Clearly this presents a major problem of interpretation as to whether the serum AFP is being produced by residual tumour or by other tissues. Follow-up of these 16 patients has shown that 4 (25%) have relapsed as indicated by progressively rising serum concentrations of AFP and clinical or radiological findings. It therefore seems likely that in a high proportion of patients with serum concentrations of AFP$>10\,ku\,l^{-1}$ treated with POMB/ACE chemotherapy, this residual immunoreactive AFP in the serum is not produced by tumour. The most frequent pattern of serial AFP concentrations is that shown in Figure 2. In this child with metastatic orchioblastoma (histologically a yolk sac teratoma) the AFP concentration fell on chemotherapy but did not reach the undetectable level for a number of months after completing therapy. In most cases the AFP concentration on POMB/ACE chemotherapy reached a plateau between 10 and $30\,\mathrm{ku\,l^{-1}}$. We have used the criterion of 12 weeks in which AFP concentrations are in this range before stopping chemotherapy provided that all other parameters of the disease are consistent with complete remission. Using these criteria there have only been 4 relapses in AFP-producing tumour in this group of 41 patients. All these 4 patients initially fell into a high-risk group as defined by an initial serum AFP concentration of $>500\,\mathrm{ku\,l^{-1}}$ prior to starting chemotherapy (Newlands *et al.*, 1983).

These results emphasise the need for a biochemical test which can discriminate between tumour-produced AFP and AFP produced by other tissues in the body. Although the AFP produced by tumour and other tissues may be immunologically cross-reactive, it may be possible to separate AFP produced by different tissues by other means. There is evidence that AFP occurs as two different molecular variants which can be separated by their affinity for concanavalin-A (Smith & Kelleher, 1973). Unfortunately assessment of this method in our department showed that it is not sensitive enough to be applied at the concentrations of AFP which present the clinical problems discussed above (T. Adams, unpublished observations). Another possibility is to use radio-crossed immunoelectrophoresis which can detect $1\,\mathrm{ku\,l^{-1}}$ AFP (Nørgaard-Pedersen & Axelsen, 1976). With this method an AFP-like substance with a gamma mobility has been identified which is measured by conventional radioimmunoassay and can reach concentrations comparable to those reported here.

We thank all the staff of the Department of Medical Oncology involved in the clinical care of these patients or in laboratory measurements and all the surgeons and radiotherapists who have referred patients to us. We also thank the Medical Research Council and the Cancer Research Campaign for support.

### References

ADINOLFI, M. (1979). Human Alphafoetoprotein 1956–78. Chapter 3, p. 165.

BAGSHAWE, K.D. (1976). Risk and prognostic factors in trophoblastic neoplasia. *Cancer*, **38**, 1373.

BAGSHAWE, K.D. & BEGENT, R.H.J. (1981). Trophoblastic tumours: clinical features and management. In *Gynecol. Oncol.*, p. 757. (Ed. Coppleson) Churchill Livingstone, Edinburgh.

BLOOMER, S.R., WALDMANN, T.A., MCINTIRE, K.R. & KLATSKIN, S. (1975). Relationship of serum α-fetoprotein to the severity and duration of illness in patients with mild hepatitis. *Gastroenterology*, **68**, 342.

CATALONA, W.J. (1979). Tumour markers in testicular cancer. *Urolog. Clin. N Am.*, **6**, 613.

ELGORT, D.A., ABELEU, G.I., LEVINA, D.M. & 4 others. (1973). Immunoradioautography test for αFP in the differential diagnosis of germogenic tumours and in the evaluation of effectiveness of their treatment. *Int. J. Cancer*, **11**, 586.

GRIGOR, K.M., DETRE, S.I., KOHN, J. & NEVILLE, A.M. (1977). Serum $\mathrm{alpha_1}$-foetoprotein levels in 153 male patients with germ cell tumours. *Br. J. Cancer*, **35**, 52.

JAVADPOUR, N. (1980). Significance of elevated serum αFP in seminoma. *Cancer*, **45**, 2166.

KURMAN, R.J., SCARDINO, P.T., MCINTIRE, K.R., WALDMANN, T.A. & JAVADPOUR, N. (1977). Cellular localization of alpha-fetoprotein and human chorionic gonadotropin in germ cell tumors of the testis using an indirect immunoperoxidase technique. *Cancer*, **40**, 2136.

MCINTIRE, K.R., VOGEL, C.L., PRINCLER, G.L. & PATEL, I.R. (1972). Serum αFP as a biochemical marker for hepato cellular carcinoma. *Cancer Res.*, **32**, 1941.

MASOPUST, J., KITHIER, K., RADL, J., KOUTECKY, J. & KOTAL, L. (1968). Occurrences of fetoprotein in patients with neoplasms and non-neoplastic diseases. *Int. J. Cancer*, **3**, 364.

NEWLANDS, E.S., BEGENT, R.H.J., KAYE, S.B., RUSTIN, G.J.S. & BAGSHAWE, K.D. (1980). Chemotherapy of advanced malignant teratomas. *Br. J. Cancer*, **42**, 378.

NEWLANDS, E.S. & BAGSHAWE, K.D. (1982). The role of VP.16–213 (Etoposide; NSC-141540) in gestational choriocarcinoma. *Cancer Chemother. Pharmacol.*, **7**, 211.

NEWLANDS, E.S., BEGENT, R.H.J., RUSTIN, G.J.S., PARKER, D. & BAGSHAWE, K.D. (1983). Further advances in the management of malignant teratomas of the testis and other sites. *Lancet*, **i**, 948.

NØRGAARD-PEDERSEN, B., ALBRECHTSEN, R. & TEILUM, G. (1975). Serum alpha-foetoprotein as a marker for endodermal sinus tumour (yolk sac tumour) or a titelline component of "teratocarcinoma". *Acta Pathol. Microbiol.* (Scand.) **83**, 573.

NØRGAARD-PEDERSEN, B. & AXELSEN, N.H. (1976). Alpha-fetoprotein-like activity in sera from patients with malignant and non-malignant disease and healthy individuals. *Clin. Chim. Acta*, **71**, 343.

PEARSON, J.C., SPAULDING, J.T. & FRIEDMAN, M.A. (1979). Testicular cancer: role of biological tumour markers. *Cancer Treat. Rev.*, **6**, 217.

SMITH, C.J. & KELLEHER, P.C. (1973). α-Fetoprotein: separation of two molecular variants by affinity chromatography with concanavallin-A-agarose. *Biochim. Biophys. Acta*, **317**, 231.

TALERMAN, A., VAN DER POMPE, W.B., HAIJE, W.G., BAGGERMAN, L. & BOEKESTEIN-TJAHJADI, H.M. (1977). Alpha-foetoprotein and carcinoembryonic antigen in germ cell neoplasms. *Br. J. Cancer*, **35**, 288.

WALDMANN, T.A. & MCINTIRE, K.R. (1974). The use of a radioimmunoassay for alpha-fetoprotein in the diagnosis of malignancy. *Cancer*, **34**, 1510.